# Assessment of Risk Factors of Noncommunicable Diseases among Semiurban Population of Kavre District, Nepal

**DOI:** 10.1155/2021/5584561

**Published:** 2021-06-07

**Authors:** Punjita Timalsina, Regina Singh

**Affiliations:** ^1^Maharajgunj Nursing Campus, Institute of Medicine, Tribhuvan University, Maharajgunj, Kathmandu 44600, Nepal; ^2^School of Nursing, Kathmandu Medical College Affiliated to Kathmandu University, Duwakot, Bhaktapur 44800, Nepal

## Abstract

Noncommunicable diseases (NCDs) are posing a great threat to mankind. Timely identification, prevention, and control of common risk factors help to reduce the burden of death from NCDs. These risk factors are also closely related to lifestyle changes. This study aimed to assess the prevalence of risk factors of NCDs among semiurban population of Kavre district. Community-based cross-sectional study design using the multistage sampling method was used to select 456 respondents. Data were collected using WHO's STEPS instruments 1 and 2. Four behavioural risk factors, i.e., current tobacco use, harmful alcohol use, physical inactivity, and inadequate servings of fruits and vegetables and two metabolic risk factors, i.e., abdominal obesity and hypertension were included in the study. The study revealed that more than one-third (36.0%; 43.0–52.2%) were current tobacco users, nearly one-sixth (15.8%; 12.7–19.4%) consumed alcohol harmfully, most of all did not have adequate servings of fruits and vegetables (95.8%; 93.6–97.3%), nearly two-thirds have abdominal obesity (62.1%; 57.5–66.4%), and more than one-fifth of population had hypertension (22.1%; 18.6–26.2%). Only 1.1% respondents were free from risk factors, while 78.5%, 46.1%, 14.5%, and 1.8% had two, three, four, and five risk factors, respectively. The co-occurrence of three or more risk factors was associated with increasing age (AOR ranging 4.7–10.9), male sex (AOR = 3.9 (2.4–6.3); *p* < 0.001), and illiterate respondents (AOR = 1.7 (1.0–2.9); *p*=0.038). The study concludes that almost all adults residing in semiurban areas of Kavre district have at least one or more risk factors, and nearly half of them have three or more risk factors. This suggests appropriate preventive approaches to be focused on younger age groups, male sex, and illiterate population to reduce the prevalence of NCDs in the near future.

## 1. Introduction

Noncommunicable diseases (NCDs) have become a medical and financial challenge in the 21st century [1, 2]. Four major NCDs (cardiovascular diseases, cancer, diabetes, and chronic respiratory diseases including asthma and COPD) are responsible for the maximum proportion of NCD deaths which share common risk factors of physical inactivity, unhealthy diet, tobacco use, and alcohol use [3–6].

Currently, NCDs accounts for more than 80% of premature deaths in developing countries [7–9]. Recent national report of Nepal NCDI Poverty Commission also revealed that the burden of NCDs has more than doubled over the past 25 years [10]. Being largely preventable, these deaths can be greatly reduced by proper intervention strategies [11].

Since most of the studies regarding NCDs have been focused either on urban or rural settings, this study was conducted to picture the prevalence of risk factors in semiurban areas of Kavre district. This study would be a prototype of villages near cities of Nepal.

## 2. Materials and Methods

A community-based cross-sectional descriptive study was carried out from December 14, 2017, to January 19, 2018, in a semiurban area of Kavre district. Approval for the implementation of the study was sought from the Institutional Review Committee (IRC) of Kathmandu University (approval number 31/17). Permission to conduct the study was obtained from District Health Office, Dhulikhel, Kavre, and then from municipalities and ward offices. The study' purpose, issues of confidentiality, anonymity, and respondents' right to withdraw from the study at any stage without notice were explained to all respondents before participation in the study. Respondents were also informed to consult health centers if any queries were encountered. Completed questionnaires were compiled and stored in a locked place by the principal researcher.

**Figure 1:**
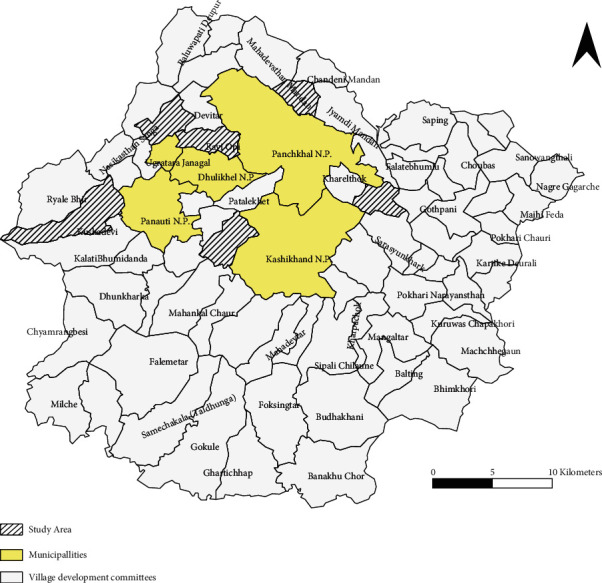
Geographical map of Kavre showing the selected semiurban areas (source: available from http://ddckavre.gov.np/en/ddc–kavre/).

Multistaged random sampling technique was used. Target population was all adults aged 18–64 years residing in semiurban areas of Kavre district. Inclusion criteria included those who were living in the place of residence for at least six months. All six municipalities of Kavre district were included in the study. From the newly declared wards of each municipality which was previously a VDC, one ward was chosen randomly (Figure 1). The number of samples to be taken from each ward was calculated following the probability proportional to size (PPS) sampling method. In the ward, a household was chosen by following systematic random sampling. Finally, at the household level, an eligible respondent was selected using a simple random sampling technique following Kish grid.

Sample size was determined by applying the following formula:

(1) $$\begin{array}{c} n=\;\frac{Z_{\alpha }^{2}pq}{d^{2}}, \end{array}$$

where *Z*_*α*_ at 95% confidence interval is 1.96, *p* is the proportion, *q* = (1–*p*), and *d* is the error taken at 20% of prevalence).

Using equation (1), we get

(2) $$\begin{array}{c} n=\;\frac{1.96^{2}\times 0.174\times 0.826}{0.0348^{2}}=455.9\text{ respondents }≅456\text{ respondents}, \end{array}$$

where *p* was taken as 17.4% as Nepal STEPS survey 2013 [4].

Thus, the sample size was 456 respondents.

According to the PPS sampling method, the following respondents were selected from each municipality:

- Panchkhal ward number 13 (12.4%) = 56 respondents out of total 461 households
- Namobuddha ward number 11 (16.1%) = 74 respondents out of total 600 households
- Dhulikhel ward number 1 (12.3%) = 56 respondents out of total 460 households
- Mandan ward number 11 (18%) = 82 respondents out of total 667 households
- Panauti ward number 2 (20.1%) = 92 respondents out of total 751 households
- Banepa ward number 4 (21.1%) = 96 respondents out of total 785 households

Data were collected using the standardized WHO STEPwise approach to Surveillance (STEPS) approaches 1 and 2, translated in Nepali language. STEP 1 data were collected by using face-to-face interview in which data were recorded in Open Data Kit (ODK) [12] toolkit. STEP 2 data included anthropometric measurements of height, weight, waist circumference, and hip circumference. Individual's height was measured without footwear and any clothing on head. Bodyweight was measured by placing a weighing machine at the smooth surface and was recorded in kilogram. Respondents were requested to wear light clothes without footwear during weighing. Waist and hip circumference measurement was conducted in a private place with a measuring tape. Waist circumference was taken at the midpoint between the lower margin of the last palpable rib and the top of the iliac crest, in the standing position without clothing and directly over the skin. The finding was recorded in centimetres. The hip circumference was taken in centimetres at maximum circumference over the buttocks horizontally. Blood pressure was measured two times at an interval of 5 minutes, and the average value of the measurements was used for the analysis. Blood pressure was taken from the left arm keeping the cuff at the same level as the heart during the procedure with elbow support. To attain the correct measurement, the respondent was not allowed to talk during the procedure, the bladder was emptied, and no coffee before and during the measurement was allowed.

After the collection of data, the data were transferred to Excel each day. SPSS version 22.0 was used for descriptive and inferential statistics. Further report was submitted to the respective wards, and a task completion letter was obtained.

## 3. Results

### 3.1. Sociodemographic Characteristics of the Respondents

Among the total 456 respondents, the mean age was 42.6 years, around two-fifths (43.0%) were male, three-fifths (57.0%) were female, nearly two-fifths were illiterate, and more than one-fifth (22.1%) belonged to the lowest quintile (Table 1).

### 3.2. Behavioural and Metabolic Risk Factors of NCDs

Nearly half of the participants (47.6%) have ever used tobacco in their lifetime, and more than one-third (36.0%) of participants were current users of tobacco. The most commonly used type of tobacco was cigarette smoking (87.8%), followed by chewing tobacco (21.3%). The mean age of initiation of tobacco among males was 18.97 years and females was 15.11 years. Nearly half of the participants (48.7%) were lifetime abstainers, while others had used alcohol sometime in their life. More than one-third of the participants (35.1%) were current drinkers. Among total participants, one-sixth were harmful users of alcohol (15.8%), while among current drinkers, nearly half (45%) were harmful alcohol users. Less than one-tenth of the participants were physically inactive (7.5%) (Table 2). The study has also revealed that females performed more physical activity compared to males. Mean hours spent in sitting on average per day was 3.96 hours. Furthermore, the majority of the participants (95.8%) took inadequate, i.e., less than 5 servings of fruits and/or vegetables on average per day. The mean number of servings of fruits and/or vegetables on average per day was 2.5 servings (Table 3).

History of hypertension was present in nearly one-sixth of the participants (17.1%), while hypertension was present in more than one-fifth of the participants (22.1%). Health risk according to abdominal obesity was also present in two-thirds (62.1%) of the participants (Table 2). The mean waist-hip ratio for males was 0.939 and for females was 0.855, which showed that the mean value is above the cutoff point for abdominal obesity (Table 3).

### 3.3. Co-Occurrence of Risk Factors

**Figure 2:**
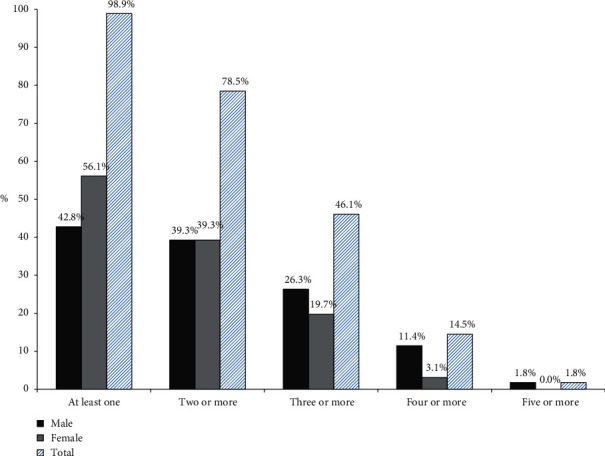
Co-occurrence of risk factors of NCDs by gender (*n* = 456).

Four biological and three behavioural risk factors were included in this study. It was found that only 1.1% of the population was totally free from these seven risk factors. Nearly half of the respondents had three or more risk factors (46.1%). After this, respondents with four or more risk factors gradually dropped to 14.5% (Figure 2). The mean number of risk factors was 2.78 risk factors (Table 3).

The co-occurrence of three or more risk factors was significantly associated with increasing age (adjusted odds ratio ranging 4.7–10.9), where males were at 3 times more risk of having combined risk factors compared to females. Similarly, illiterates had 1.7 times more risk of having combined risk factors compared to literates (Table 4).

## 4. Discussion

The rate of current tobacco use is double than national data, i.e., 18.5% but lower than NDHS surveillance data (39.8%) [4, 13]. This might be due to inclusion of all age groups in NDHS survey and only adults in STEPS survey. Similar study in squatter of Kathmandu valley reported 39.81% current drinkers [14]. Another study at Sindhupalchowk revealed prevalence of alcohol drinking to be 62.9% [15]. These findings are similar to findings of this study.

The study among adults of Duwakot showed low physical activity among 43.3% [16]. Another study at Kathmandu also reported low physical activity among 17.6% [17]. Few participants with low physical activity in this study might be due to semiurban area of Kavre district still being agricultural region which demands more physical activity which is correlated with national STEPS survey, which revealed low physical activity only in 3.5% of participants [4].

STEPS survey also revealed overweight among 17.7% and obesity among 4% of study participants [4]. This variation might be due to inclusion of rural regions also where physical activity is adequate. Demographic survey of Nepal also revealed overweight among 22% female and 17% of males [13]. These findings are similar to findings from this study. Mean waist-hip ratio was 0.9 for both male and female in national STEPS survey [4]. Study in Bharatpur revealed abdominal obesity among 81.7% of males and 94.1% of females [18]. This variation might be due to Bharatpur being more urban region.

STEPS survey concluded only 0.4% of sample to be free from risk factors [4]. Another evidence of cumulative risk factors in Uganda revealed that most participants exhibited at least two risk factors supported by mean number of risk factor of our study [19]. These all findings suggest that burden of combined risk factors of noncommunicable is in increasing trend. Similar study in Bangladesh regarding clustering of three or more risk factors revealed association of clustering of risk factors with age, male sex, and educational level [8]. This supports the association found in our study.

## 5. Conclusions

The prevalence of risk factors of noncommunicable disease is high in semiurban setting of Kavre district. Additionally, there exist gender differences in the prevalence of risk factors, with more risk factors occurring in males compared to females. Moreover, the risk factor is more among illiterate than literate. Therefore, it is necessary to minimize the burden of growing noncommunicable disease epidemic in semiurban areas of Kavre district. This can be done by conducting awareness programs regarding NCD risk factors continuously to bring behaviour change. This should also be focused on early age because the risk of noncommunicable diseases is fairly low in this age group. Since the initiation of smoking and alcohol use is at adolescence, smoking cessation and awareness campaigns should be targeted to these groups to prevent the adoption of risk behaviours. In addition, persons could be informed of body mass index and waist circumference and avoid pot belly. Additionally, appropriate plans can be drafted in new urban and federal health policies.

## Figures and Tables

**Table 1:** Sociodemographic characteristics of the respondents (*n* = 456).

Variables	Male, *n* (%)	Female, *n* (%)	Total, *n* (%)	*P* value
Age group (in years)				
18–24	18 (9.2)	34 (13.1)	52 (11.4)	0.439
25–34	30 (15.3)	45 (17.3)	75 (16.5)	
35–44	51 (26.0)	74 (28.4)	125 (27.4)	
45–54	50 (25.5)	54 (20.8)	104 (22.8)	
55–64	47 (24.0)	53 (20.4)	100 (21.9)	
Age (in years), mean ± SD	44.0 ± 12.6	41.5 ± 13.1	42.6 ± 12.9	0.434

Gender	196 (43.0)	260 (57.0)	456 (100.0)	—

Education				
Illiterate	34 (17.3)	144 (55.4)	178 (39.0)	<0.001
Literate	162 (82.7)	116 (44.6)	278 (61.0)	

Monthly household income by quintile groups				
Fifth quintile	36 (18.4)	36 (13.8)	72 (15.8)	0.770
Fourth quintile	55 (28.1)	74 (28.5)	129 (28.3)	
Third quintile	42 (21.4)	60 (23.1)	102 (22.4)	
Second quintile	21 (10.7)	31 (11.9)	52 (11.4)	
First quintile	42 (21.4)	59 (22.7)	101 (22.1)	

**Table 2:** Behavioural and metabolic risk factors of NCDs (*n* = 456).

Risk factors	*n* (%)	95% CI
*Behavioural risk factors*		
Tobacco use		
Ever users	217 (47.6)	43.0–52.2
Current users	164 (36.0)	31.7–40.5
Daily users	161 (35.3)	31.1–39.8

Alcohol use		
Lifetime abstainers	222 (48.7)	44.1–53.3
Past 12 months abstainer	54 (11.8)	9.2–15.1
Drank only in past 12 months	20 (4.4)	2.9–6.7
Current drinkers	160 (35.1)	30.8–39.6

Among current drinkers (*n* = 160)		
Category I drinker	55 (34.4)	27.5–42.0
Category II drinker	33 (20.6)	15.1–27.5
Category III drinker/harmful alcohol use	72 (45.0)	37.5–52.7
Inadequate servings of fruits and vegetables	437 (95.8)	93.6–97.3

Level of physical activity		
High	383 (84.0)	80.3–87.1
Moderate	39 (8.6)	6.3–11.5
Low	34 (7.5)	5.4–10.2

Metabolic risk factors		
Raised blood pressure	101 (22.1)	18.6–26.2
Abdominal obesity (waist-hip ratio)	283 (62.1)	57.2–66.3

**Table 3:** Summary of risk factors of NCDs (*n* = 456).

Risk factors	Male	Female	Total	*P* value
	Median (IQR)	Median (IQR)	Median (IQR)	
Standard drinks, number/occasion	5.7 (2.6–12.8)	3.25 (1.4–5.85)	5.3 (2.0–10.6)	0.011
Fruits/vegetables, servings/day	2 (2-3)	2 (2-3)	2 (2-3)	0.632
Physical activity, MET minutes/week	7340 (2405–13860)	8400 (5400–12600)	8400 (5040–12870)	0.127
Body mass index	23.8 (21.9–26.5)	23.8 (21.4–27.2)	23.8 (21.6–27.0)	0.655
Waist circumference (cm)	87 (79.2–94.0)	78.5 (70–88)	82 (82–90)	<0.001
Waist-hip ratio	0.94 (0.89–0.98)	0.86 (0.80–0.91)	0.89 (0.83–0.94)	<0.001
BP, systolic (mm of Hg)	110 (100–130)	105 (95–120)	110 (95–120)	0.001
BP, diastolic (mm of Hg)	75 (66.25–85)	70 (60–80)	70 (65–80)	0.016

**Table 4:** Association of co-occurrence of three or more risk factors with sociodemographic variables.

Sociodemographic variables	OR (95% CI)	*P* value	AOR (95% CI)	*P* value
Age				
18–24 years	Reference		Reference	
25–34 years	5.0 (1.8–14.1)	0.002	4.7 (1.6–13.7)	0.005
35–44 years	7.4 (2.7–19.8)	<0.001	6.6 (2.3–18.5)	<0.001
45–54 years	13.9 (5.1–37.8)	<0.001	10.9 (3.8–31.3)	<0.001
55–64 years	15.3 (5.6–42.0)	<0.001	10.9 (3.7–32.1)	<0.001

Sex				
Female	Reference		Reference	
Male	3.0 (2.0–4.4)	<0.001	3.9 (2.4–6.3)	<0.001

Educational status				
Literate	Reference		Reference	
Illiterate	1.4 (0.9–2.1)	0.054	1.7 (1.0–2.9)	0.038

Household income				
Richest (20%)	Reference		Reference	
Second quintile	0.7 (0.4–1.3)	0.243	0.7 (0.3–1.4)	0.309
Third quintile	0.5 (0.2–0.9)	0.031	0.5 (0.3–1.0)	0.056
Fourth quintile	0.8 (0.4–1.7)	0.606	0.9 (0.4–1.9)	0.767
Poorest (20%)	0.9 (0.5–1.8)	0.969	0.9 (0.5–1.8)	0.813

## Data Availability

The datasets used and/or analysed during this study are available from the corresponding author upon request.
